# Phosgene-induced acute lung injury (ALI): differences from chlorine-induced ALI and attempts to translate toxicology to clinical medicine

**DOI:** 10.1186/s40169-017-0149-2

**Published:** 2017-06-02

**Authors:** Wenli Li, Juergen Pauluhn

**Affiliations:** 1 4th Department of Toxicology, Fourth Military Medical University, No. 169 Changle West Road, Xi’an, 710032 Shaanxi Province China; 2 Covestro Deutschland AG, Global Phosgene Steering Group, K9, 565, 51365 Leverkusen, Germany

**Keywords:** Acute lung injury, Nociceptive sensory reflexes, Cardiopulmonary function, Biomarkers in expired gas

## Abstract

**Background:**

Phosgene (carbonyl dichloride) gas is an indispensable chemical inter-mediate used in numerous industrial processes. There is no clear consensus as to its time- and inhaled-dose-dependent etiopathologies and associated preventive or therapeutic treatment strategies.

**Methods:**

Cardiopulmonary function was examined in rats exposed by inhalation to the alveolar irritant phosgene or to the airway irritant chlorine during and following exposure. Terminal measurements focused on hematology, protein extravasation in bronchoalveolar lavage (BAL), and increased lung weight. Noninvasive diagnostic and prognostic endpoints in exhaled breath (carbon dioxide and nitric oxide) were used to detect the clinically occult stage of pulmonary edema.

**Results:**

The first event observed in rats following high but sublethal acute exposure to phosgene was the stimulation of alveolar nociceptive vagal receptors. This afferent stimulation resulted in dramatic changes in cardiopulmonary functions, ventilation: perfusion imbalances, and progressive pulmonary edema and phospholipoproteinosis. Hematology revealed hemoconcentration to be an early marker of pulmonary edema and fibrin as a discriminating endpoint that was positive for the airway irritant chlorine and negative for the alveolar irritant phosgene.

**Conclusions:**

The application of each gas produced typical ALI/ARDS (acute lung injury/acute respiratory distress syndrome) characteristics. Phosgene-induced ALI showed evidence of persistent apnea periods, bradycardia, and shifts of vascular fluid from the peripheral to the pulmonary circulation. Carbon dioxide in expired gas was suggestive of increased ventilation dead space and appeared to be a harbinger of progressively developing lung edema. Treatment with the iNOS inhibitor aminoguanidine aerosol by inhalation reduced the severity of phosgene-induced ALI when applied at low dose-rates. Symptomatic treatment regimens were considered inferior to causal modes of treatment.

## Background

Phosgene (carbonyl dichloride) gas is an indispensable chemical intermediate used in numerous industrial processes at a global annual production scale of approximately 15 million metric tons. In contrast to most irritant gases, phosgene is poorly soluble in water, reacts by acylation with nucleophilic moieties, and causes pulmonary edema with a typical clinical “latency” or, more appropriately described, an clinically “occult” period. At such production scales, phosgene is commonly produced on demand and solvent-free by high-yield catalyzed gas-phase reactions of carbon monoxide and chlorine. Phosgene is an essential building block for the synthesis of isocyanates, which serve as monomers for paints, coatings, insulating foams, and special-use chemicals, to mention but a few.

Phosgene and chlorine are most notorious as early chemical warfare agents, first used militarily in 1915 during World War I (WWI). For operational purposes, they were denoted as either G52 (during WWI) or CG (WWI and later) [1–8]. A comprehensive overview of warfare agents, including phosgene, has been published [9–13]. Within military communities [both United States (US) and non-US], phosgene has been the subject of many toxicological studies, human toxicity estimates (ranging from threshold mild effects to lethality), and other general reviews and commentaries [12–15].

Therapeutic approaches have traditionally been based on hypothetical pathways elucidated through in vivo research on rodents. Putative mechanisms of phosgene-induced ALI range from direct interaction and deterioration of lung surfactants and associated changes in lung mechanics [16, 17] to free radical attacks on neuronal, endothelial and epithelial cells, resulting in tissue destruction and mediator release [18, 19]. Canine, ovine, and porcine models have been used when studying the cardiopulmonary and hematological effects of methods used in humans. These models included protective ventilation in terminally anesthetized animals and pharmacological interventions such as steroids and bronchodilators [18–26]. Many of the drugs examined in these studies were shown to be ineffective in follow-up proof-of-principle studies. Concurrent with the change in paradigms for treating ALI/ARDS (acute lung injury/acute respiratory distress syndrome), the emphasis of more recent research has shifted from treating to preventing acute lung injury using triage-based preemptive, personalized ventilator strategies applied to maintain normal lung function in patients at high risk [27–32]. Supportive treatment commonly includes increased fractional concentrations of FiO_2_, the use of PEEP, and physical rest. However, preventive treatment of phosgene-induced ALI with ‘injury-adjusted’ partial pressures of O_2_ and PEEP settings has not been systematically investigated. Countermeasures were further complicated because ‘trial and error’ types of treatment strategies were already applied in the absence of indications and robust diagnostic tools that would have given prognostic guidance to clinicians.

Mechanism-based causal countermeasures require an in-depth understanding of the adverse outcome pathway (AOP), including its concentration × time relationship, initiating and amplifying the respective life-threatening condition. While past approaches focused on pharmacological interventions to mitigate phosgene-induced pulmonary edema, the focus of the research described in this paper was to better characterize the onset and interrelationships of early types of physiological dysregulation as initiating events causing progressively developing pulmonary edema. Unlike other, more water-soluble irritant gases, such as HCl or chlorine, potentially lethal exposure to phosgene may not subjectively perceived as such. Thus, clinically occult lung edema might occur within the asymptomatic period of patients, which then changes precipitously with time after exposure, leading to respiratory failure and death. The odor threshold for phosgene is significantly higher than current inhalation exposure limits [5, 33–35]. Thus, odor or sensory irritation provides insufficient warning or clinical evidence of hazardous exposure doses.

Despite overwhelming evidence from both toxicological and medical research, even recently published papers often begin with the following statement: “*Owing to its poor water solubility, one of the hallmarks of phosgene toxicity is an unpredictable asymptomatic latent phase before the development of noncardiogenic pulmonary edema*”. Notably, the “latent” or, more appropriately phrased, clinically “occult” period of phosgene poisoning is the largely asymptomatic interval between exposure and the onset of edema by conventional methods. This definition is a fallacy since the incipient anatomic and pathophysiologic lung injury occurs with exposure and steadily progresses until sufficiently severe to become phenotypically detectable. Its occurrence follows a typical reciprocal inhaled concentration x time relationship. At exposure intensities within the range of 300–500 ppm × min, pulmonary edema occurs few hours post-exposure, followed by lethality ≈12–24 h later. At much higher exposure intensities, this period may becomes markedly shorter [35, 36]. Delayed mortality was also observed in experimental models of phosgene examined 80 years ago [24]; however, it was absent in more recent studies [37, 38]. Accounting for the fact that the more recent industrial production of phosgene is by catalytic reaction of the high-purity gases anhydrous chlorine and carbon monoxide, the presence of irritant impurities causing airway injury can be ruled out. The largest-scale human exposures to chlorine occurred during World War I, when the gas was used as a chemical weapon. Chlorine-induced oxidative injury and normal repair of the respiratory epithelium of the airways was critical to preventing the long-term pulmonary pathology that can occur following acute injury [39, 40].

This review discusses the most salient findings from toxicological and pharmacological research on rats and dogs over a period of one decade [17, 20, 37, 38, 41–50]. The objective of this project was not only to develop inhalation exposure systems to expose rats and dogs to phosgene under highly controlled conditions and similar modes of exposure [20, 33, 37, 38, 49, 51] but also to study the early physiological events involved in phosgene-induced ALI, including options for causal and preventive treatment strategies. This process included the identification of early biomarkers of pulmonary injury that predict life-threatening pulmonary edema. Although most of the mechanistic endpoints were invasive in nature, emphasis was also directed toward non-invasive diagnostic methods that are translatable to clinical practice. One of the ancillary objectives of this work was to search for diagnostic tools to provide integrated information as to how triage and countermeasures could be structured for patients exposed to mixtures of phosgene and chlorine, a precursor of phosgene. To achieve these objectives, methods used in toxicology must be translatable to those used in humans.

## Inhalation method—rats

Rats were exposed to phosgene (COCl_2_) using a *directed*-*flow nose*-*only inhalation principle* [33, 37, 51]. Current testing guidelines give preference to this mode of inhalation exposure [52]. Certified gas standards with specified stability in synthetic air were used in all studies. The gas was contained in 10 L cylinders @150 bar. The volume-to-mass conversion factor for phosgene is 1 ppm = 4.1 mg/m^3^. Throughout all studies, the exposure period was 30 min. Air flow, temperature, and humidity measurements in the inhalation chamber utilized a computerized data acquisition and control system. The exposure conditions were adjusted to maintain an airflow rate of 0.75 L/min per rat, which is threefold higher than the respiratory minute ventilation of the rat. Under the given conditions, inhalation chamber state–state was attained within the first minute of exposure. The analytical concentrations from the inhalation chamber were in agreement with the nominally calculated concentrations, which were targeted at 30–35 mg phosgene/m^3^ (≈1000 mg/m^3^ × min or ≈250 ppm × min). In studies aimed at toxicological endpoints, the characterization of test atmospheres utilized OSHA method no. 61 (http://www.osha-slc.gov/dts/sltc/methods/organic/org061/org061.html) using gas bubblers filled with a toluenic solution of the trapping agent 2-hydroxymethyl-piperidine (2-HMP). The resultant analyte was then analyzed by gas chromatography. For mechanistic and intervention studies, actual concentrations were determined in real time using a calibrated Gasmet Dx-4000 FT-IR (Fourier transform infrared spectroscopy) gas analysis system (for details see http://www.gasmet.com/images/tiedostot/product-downloads/Gasmet_DX4000_Technical_Data_(v1.6).pdf). The spatial homogeneity and temporal stability of phosgene in exposure atmospheres were controlled in real time [37].

Rats exposed first to phosgene and then to the aerosolized drug aminoguanidine were exposed nose-only, similar to phosgene [44], or in a small whole-body inhalation chamber with dynamic air flow and aerosol generation at targeted and analytically verified concentrations of ≈300 mg drug/m^3^. The comparison of nose-only and whole-body exposed rats served the purpose of judging the impact of “psychological immobilization stress” and associated cardiovascular stimulation due to restraint relative to non-immobilized, whole-body-exposed rats. Under such exposure conditions, the inhaled dose rate of drug is equivalent to ≈16 mg/kg-rat/hour.

Rats were anesthetized by intraperitoneal injection of pentobarbitone, and blood was collected from the left ventricle at sacrifice. Animals were exsanguinated by severing the abdominal aorta. Then, the excised lungs were weighed, and bronchoalveolar lavage fluid (BALF) was obtained as detailed elsewhere [38, 42].

## Inhalation methods—larger animals

Details of the head-only chamber used for dog inhalation studies have been published elsewhere [17, 20]. This mode of exposure to phosgene differed from those of other authors using larger animals. For reference, the reader is advised to consult more detailed reviews and papers on larger animals used for studies with phosgene [9, 21, 22, 24, 53]. Larger inhalation chambers may be useful to accommodate larger animals or larger numbers of small animals. For technical reasons and the difficulty of generating homogeneous exposure atmospheres at short exposure durations, a more human-like exposure mode and regimen may jeopardize the outcome of the study due to technical shortcomings. Especially for pharmaceutical countermeasures delivered by the inhalation route, particular attention must be paid to maintaining similarities of the dosing regimen used in the bioassay with that used in humans. Otherwise, meaningful interspecies extrapolations and dosimetric adjustments are hampered. The endotracheal administration of phosgene and inhalation drugs may overcome some of these difficulties; however, due to the numerous manipulations required, this may cause additional uncertainties concerning the inhaled dose. Compared to small animals, dogs and pigs offer the advantage that these species have also been used in pre-clinical studies of inhalation pharmaceuticals. Their breathing physiology is closer to that of humans than that of rodents. The size and anatomy of their lungs, including the large tracheobronchial tree and vascular architecture, make it possible to use the same equipment as used in intensive care units (ICUs). Thus, when making judgements as to the extent to which a small or large animal model delivers the most significant information for human risk assessment, numerous methodological and species-specific factors must be considered. These factors include that the exposure and treatment of larger animals using endotracheal tubes and terminal anesthesia may not only complicate translation dosimetry but may also affect reflex-mediated responses to exposure and injury.

### Inhalation dosimetry

Experimental inhalation studies with irritant gases cannot be considered as a “one-size-fits-all” approach. In case the most critical effect occurs in the lower airways of the respiratory tract, water solubility and chemical reactivity produce a marked concentration-dependent anterior–posterior gradient of injury within the tract. Depending on the concentration inhaled, the irritant gas will be scrubbed in the upper airways of obligate nasal-breathing rodents, whereas it may reach the lower airways in oronasally breathing humans. Hence, the sites of retention and injury may differ appreciably in relation any chosen concentration × time (exposure duration) relationship. Haber’s rule, “C^n^ × t = *constant effect*” with n = 1, is fulfilled for phosgene but deviates for other gases. The inhaled dose (Cxt) may vary appreciably across species with different respiratory minute volumes. Animal models of the past attempted to overcome this rodent-specific shortcoming by delivering test agents into the lung by endotracheal tubes. In doing so, the retained dose of the gas within the tract may possibly be more human-like at first glance; however, the distribution of the inhaled dose relative to the inspired volume and concentration gradient within the tract remains uncertain. Anesthesia, dead-space volumes and rebreathing increase the dosimetric uncertainties as well. Accordingly, animal models need to be dosimetrically adjusted to a ‘human-equivalent dose’ to produce the same profile of lung injury. This process may require higher equivalent concentrations in rodents to overcome the loss of agent within the upper airways.

Notably, phosgene is an alveolar irritant with negligible scrubbing of gas within the airways. Thus, the gas penetrates the lower respiratory tract independently of concentration, and injury becomes solely dependent on the inhaled Cxt but not C alone. On the other hand, chlorine is a water-soluble gas that is retained throughout the airways that also becomes an alveolar irritant at higher concentrations. Thus, the injury patterns of chlorine change with both C and t. Thus, while C determines the depth of lung penetration and airway retention, the related Cxt determines the dose and severity of injury at this site of retention [33, 45, 50]. High-risk patients from accidental occupational exposure to phosgene can readily be identified by the mandatorily worn Cxt-based dosimeter (“Phosgene badge”).

## Mechanisms of toxicity and hypotheses

The incipient pathogenesis of phosgene-induced ALI/ARDS starts with loss of surfactant function, which is caused by direct chemical reactions of surfactant with phosgene [33, 45]. This loss then may lead to a heterogeneous collapse of alveoli and ensuing changes in lung mechanics. Although additional mechanisms cannot entirely be excluded, the alteration of the Starling fluid flux equation, e.g., due to increased interstitial pressure, further destabilizes the alveoli. Accordingly, the time for treatment is before, not after, edema has appeared, as previously concluded by Coman et al. [24] 80 years ago.

Such a series of events calls for preventive, rather than therapeutic, modes of treatment. However, any proposed therapies targeting prevention or early treatment of lung injury prior to respiratory failure require specialized diagnostic tools to identify early at-risk patients who will actually develop ALI/ARDS. Progress in specific treatments for ALI/ARDS beyond the lung-protective strategies of mechanical ventilation and conservative fluid management needs to be realized [31, 54]. Hence, the currently instituted reactive rather than proactive approaches regarding lung protection should be refocused on preventing the progression of worsening lung injury with time elapsed post-exposure rather than attempting to treat respiratory failure that becomes increasingly refractory to any type of treatment.

### Reactivity of phosgene leading to alveolar irritation

The low solubility of phosgene in water enhances its acute toxicity by allowing the inhaled gas to penetrate into the alveolar spaces without any appreciable losses of this gas in the extra- and intrathoracic airways. The physicochemical properties of phosgene also preclude any spontaneous hydrolysis within this microenvironment because the reaction of phosgene with water is much slower than the reaction with the more nucleophilic chemical moieties –NH_2_, –OH, and –SH. Accordingly, as a strong electrophile, phosgene may avidly react with such nucleophilic moieties of peptides and proteins present in this environment [35, 55]. The affinity of phosgene for thiol (–SH)-bearing molecules, such as cysteine (Cys) and glutathione (GSH), is sufficiently high to successfully compete with water. The inhibition of –SH enzymes produced by phosgene is irreversible and was shown to be ineffective target for the mitigating phosgene poisoning [56]. Hence, such interactions make it possible that phosgene can specifically affect homeostatic redox, protein-anti-protein and other equilibria. Reversibility can be achieved only by the re-synthesis of protein.

Rats acutely exposed to phosgene at 600 mg/m^3^ for 1.5 min (225 ppm × min) [49] were exposed to the aerosolized nucleophiles hexamethylenetetramine (HMT), Cys, and GSH for 5 or 15 min at 1 mg/L air. Cys and GSH exhibited anti-oxidant properties in addition to their nucleophilic mode of action. The efficacy of treatment was judged by protein concentrations in BALF collected at the climax of the phosgene-induced lung edema one day post-exposure [33, 37, 38, 49]. Despite the use of optimized aerosolization to maximize the penetration of aerosolized drugs into the lower lung, none of the chosen nucleophiles mitigated the phosgene-induced ALI. This finding suggests that the reaction products of phosgene could not be reversed even when instant inhalation treatment was applied. Likewise, despite the direct administration of aerosolized anti-oxidants with phosgene-scavenging properties, all treatments were ineffective [49, 57]. To the contrary, the prophylactic parenteral or oral administration of nucleophiles, such as HMT [49, 58] and cysteine esters [59], was demonstrated to provide measureable protection when given before exposure to phosgene.

Consistent with the findings detailed above, these compounds did not demonstrate any protection when given after exposure to phosgene [58–61]. This outcome provides indirect evidence that the acylation of nucleophilic moieties of surfactant appears to be more critical than any putative release of HCl. This overall interpretation is supported by the LCt_50_ of phosgene in acutely exposed rats (1741 mg/m^3^ × min) [37], which is orders of magnitude lower than that of HCl gas (211,545 mg/m^3^ × min) [33]. In this context, it is important to recall that the GSH content in human airway lining fluids is 140-fold higher than in plasma and that this most important protective nucleophile is known to be of inferior significance in rats, whose anti-oxidant status is predominantly controlled by ascorbic acid [62].

### Mechanisms of phosgene leading to lethal lung edema

**Fig. 1:**
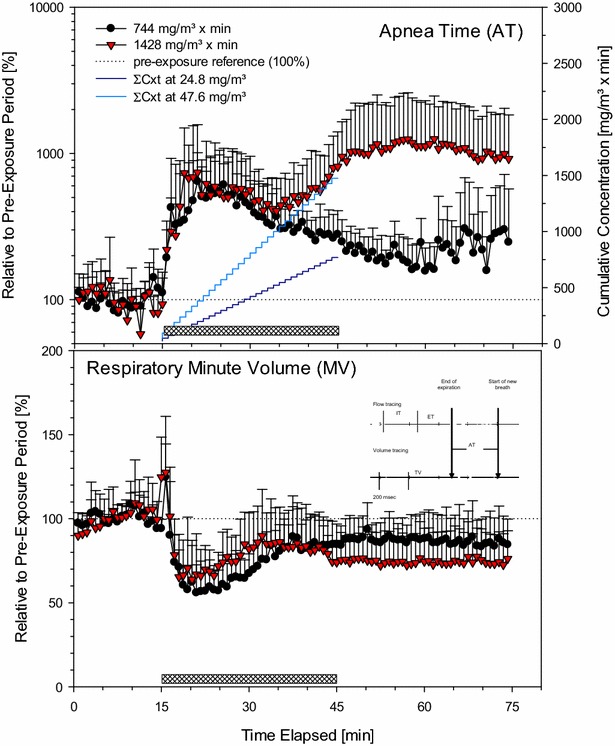
Analysis of respiratory patterns focused on AT and MV. Measurements were made in conscious, spontaneously breathing restrained rats placed in nose-only volume-displacement plethysmographs (pressure = const.). Animals were exposed in three subsequent steps to air (15-min, pre-exposure baseline data), phosgene (30-min, hatched *bar*), and air again (30-min, recovery). Data averaged during time-periods of 45-sec and represent means + SDs from eight simultaneously exposed rats/group. The insert given in the *lower panel* shows two analog tracings that represent flow-derived (*top*) and integrated volume-derived (*bottom*) changes, respectively. X-axis: 200 ms/tick. The breath structure is characterized by three phases: IT, ET and AT. These phases are used to distinguish between *upper* airway respiratory tract irritants (bradypnea period between IT and ET; not observed) and *lower* respiratory tract irritants (apnea period between end of ET and start of new breath). Such pauses do not occur in air only exposed rats. The integrated volume over flow of one breath was the tidal volume (V_T_). The product of number of breaths (respiratory rate) × V_T_ was taken as the respiratory minute volume. The stepped *curves* represent the accumulated Cxt over the duration of exposure to phosgene

If the buffering capacity of the surfactant layer toward phosgene is exhausted, direct contact and damage of tissue cannot be excluded. From that aspect, phosgene gas is expected to produce a clear-cut, precipitous toxicity restricted to the alveolar region [37, 38]. If the protective layer of surfactant becomes dysfunctional or deteriorated, alveolar collapse and ventilation: perfusion disturbances may ensue as the most likely outcome. This outcome is the first step toward increased dead space (intact alveolar ventilation but compromised perfusion) or shunt (intact perfusion but compromised alveolar ventilation). This situation is further complicated by the vasoactive and pro-inflammatory mediators released by damaged and necrotic alveolar macrophages, followed by increased accumulation of fluids in the septal interstitium (Fig. 1). The resultant congestion and loss of elastic recoil may additionally affect alveolar compliance. Pulmonary vessels and airways may become mechanically compressed, which overrules patency by tonus.

The direct interaction of phosgene with nerve endings from vagal C-fibers may add additional complexity with increasing loss of fine-tuned neurovascular control. The local stimulation of mechanosensory nerves may additionally affect the cardio-pulmonary synchronization and cardiovascular disturbances that contribute to hemodynamic changes and imbalances, leading to the translocation of fluids from the peripheral into the pulmonary circulation.

Despite this complexity occurring at near lethal Cxts, single and repeated subchronic 90-day inhalation studies of rats with 6 h/day exposure 5 times/week demonstrated that the chronic effects of phosgene gas appear to be contingent on “acute-on-chronic” localized effects. Essentially identical NOAELs were observed independent of whether the duration of exposure was acute or subchronic [33, 63]. In contrast to more water-soluble irritant gases, airway toxicity or delayed-onset types of inhalation toxicity (e.g., *obliterating bronchiolitis*) were not observed in the more recent animal models of phosgene [33, 37, 38].

Although considered an irritant gas due to its high water solubility, chlorine (Cl_2_) readily partitions into the fluids lining the airways. Once Cl_2_ is dissolved into the fluids lining the airways, epithelial damage and desquamation occur as a result of oxidative injury. This may occur with exposure to Cl_2_, and further damage to the epithelium may occur with the migration and activation of inflammatory cells. Repair of the airway epithelium following Cl_2_-induced injury may not necessarily restore normal structure and function, as evidenced by subepithelial fibrosis and excessive mucous hyperplasia. The oxidative mechanism of toxicity caused by chlorine is less specific than that attributed to the more selective electrophilic reactivity of phosgene. Hence, while chlorine may elicit different patterns of injury (airway injury with or without alveolar damage) depending on the inhaled dose and concentration, phosgene damage is largely independent on concentration and restricted to alveolar injury. Thus, anti-inflammatory countermeasures can be anticipated to be efficacious for chlorine-induced lung injuries, whereas they can be anticipated to be ineffective or even contraindicated for phosgene.

## Experimental studies

### Lethality thresholds in experimental animals and humans

The non-lethal time-adjusted threshold concentration (LCt_01_) in rats was ≈1000 mg/m^3^ × min (225 ppm × min) [37]. The respective value estimated for humans was ≈300 ppm × min (≈1200 mg/m^3^ × min) [64]. Thus, with regard to this acute point of departure (POD), there is remarkable similarity between rats and humans [5, 33]. If not mentioned otherwise, the mechanistic and intervention studies addressed in this paper utilized a ≈1000 ± 50 mg/m^3^ × min delivery over a 30-min exposure period. Interventions commenced shortly after exposure. Efficacy was judged by measurements of BAL and lung weight 1 day post-exposure, i.e., the climax of pulmonary edema.

### Stimulation of sensory nerves in the lower respiratory tract

Acute lung injury in rats caused by the inhalation of phosgene gas was shown to elicit changes in cardiopulmonary functions, including changes in the control of breathing that preceded pulmonary edema. These dysregulated functions appeared to be associated with multiple factors originating from local neurogenic, pharmacological, and mechanical changes suitable to further orchestrate any centrally controlled cardiovascular function. Early studies in dogs [10, 65] reported that the heart rate fell precipitously with exposure to phosgene and then slowly rose to the initial value or higher. Small-animal bioassays were devised to more systematically study these types of phosgene-induced time-course relationships.

Rats with nose-only exposure to phosgene exhibited an instant ≈50% depression in respiratory minute volumes on volume-displacement plethysmographs when exposed to 744 and 1428 mg phosgene/m^3^ × min [37]. Partial recovery occurred shortly after the nadir of this response was reached (Fig. 1). However, recording the apnea time (AT), the period between two breathing cycles (see insert of Fig. 1), revealed a rapid ≈fivefold increase in AT. At exposure concentrations of both 24.8 and 47.6 mg/m^3^, a similar increase occurred up to ≈10 min of exposure, followed by a decrease toward normal breathing at 24.8 mg/m^3^. At 47.6 mg/m^3^, the opposite occurred when a cumulative exposure dose of ≈1000 mg/m^3^ × min was attained (stepped line in Fig. 1, upper panel). The POD from reflexively related changes suggestive of progressive loss in the control of pulmonary mechanics coincided with the LCt_01_ threshold occurring 10–20 h post-exposure.

**Fig. 2:**
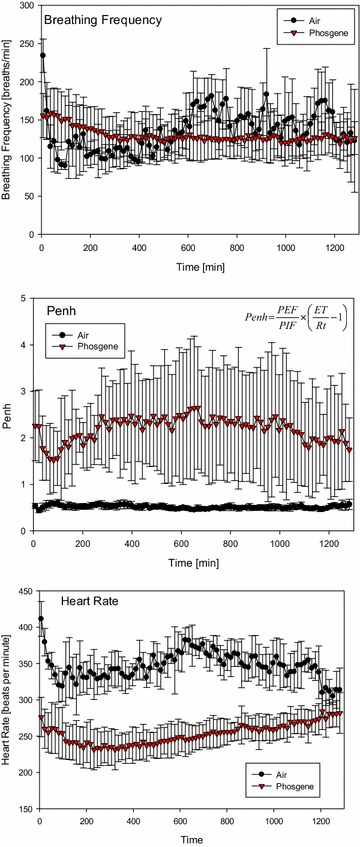
Analysis of cardiopulmonary functions of conscious, spontaneously breathing, unrestrained rats placed in whole-body barometric flow-plethysmographs (food and water accessible) from the end of exposure to phosgene until the next morning during a data collection-period of 20 h. Measurements focused on respiratory rate, enhanced pause (Penh) and heart rate using radiotelemetry transmitters intra-peritoneally placed in a single lead II configuration (DSI PhysioTel^®^ Implantable Telemetry System). Data represent means ± SD from eight rats/group (averaged during collection periods of 15 min). Insert (*mid panel*): Penh is a unitless index that was used to evaluate changes in the shape of the airflow patterns as index of changes in the control of breathing secondary to ALI

In contrast to volume-displacement plethysmograph measurements performed simultaneous to phosgene inhalation exposure (Fig. 1), equally exposed rats were evaluated for changes in the shape of the airflow pattern entering and leaving a whole-body-flow plethysmograph as the animal breathed (Fig. 2). The experimental arrangement applied allowed contemporaneous measurements of both pulmonary and cardiac functions in freely moving, habituated rats [42, 47]. Data collection started shortly after exposure to phosgene or chlorine and continued for up to approximately 20 h. The most salient changes in pulmonary function were indicated by increased enhanced pause (Penh), a dimensionless index. This index is sensitive to changes in the breathing cycle, especially the prolongation of apnea periods occurring during the expiratory phase [48]. Cyclically changing apnea periods occurred periodically and increased the inter-animal variability in phosgene-exposed rats, but not in normally breathing control rats (Fig. 2). The physiological significance of the use of Penh to assess pulmonary function has been challenged [53, 66–73]. The mechanical properties of the lungs are characterized by their main determinants, resistance and elastance. Resistance is the ratio of the pressure to the flow, while elastance is the ratio of the pressure to the volume. Therefore, to calculate either of these quantities, two signals need to be measured: pressure and either flow or volume. Penh is based on only a single time-varying signal, the pressure inside a plethysmograph; thus, it simply does not convey the information required to provide a valid estimate of lung mechanics. At best, Penh represents a type of nonspecific reflection of the pattern of breathing [73].

While entirely in agreement with the conclusions of these authors, Penh was used as an all-integrating endpoint mirroring the apparent loss in the elastance of pulmonary parenchymal tissue with ensuing changes in the control of breathing. With increased elastic resistance of the lung by injury, the respiratory pattern needs to be adjusted to minimize the mechanical work of breathing to overcome increased elastic tissue resistance. This interpretation was further substantiated by a strong correlation between increased lung weight and increased Penh induced by a pulmonary irritant aerosol [48, 74]. Thus, despite its known shortcomings, Penh appears to integrate several physiological endpoints in a wholly non-invasive manner so that non-specific functional changes can be readily identified without restraint of rats over long data-collection periods. Although specific pathophysiological effects cannot be revealed by this index, Penh appears to reflect the integrated adaption of breathing to changes in lung mechanics.

Typical ECG tracings of rats exposed to air (control) or phosgene have been published elsewhere [42]. Due to the lack of a common isoelectric baseline, each cycle was established on its own reference level. One of the unique electrophysiological characteristics of the rat ECG is the absence of a Q wave and the lack of an isoelectric ST-segment. Consequently, there is no clear separation between the QRS complex and the T-wave. The change in heart rate (sinus bradycardia), which was among the most prominent findings distinguishing phosgene-exposed rats from controls, attained a nadir approximately 4 h post-exposure (Fig. 2). The time-course changes observed in control rats were attributed to the rats’ nocturnally increasing activity (nycthemeral biorhythm). Other cardiological changes that were observed were considered to be adaptive and secondary to bradycardia, i.e., functional changes typical of afferent pulmonary C fiber J receptor stimulation (increased AT). Continued bradycardia after exposure to phosgene and other signs typical of excessive parasympathetic tone have also been observed in humans [75, 76]. Although vagotomy and parasympatholytic drugs (atropine) prevented or abolished the neurogenic etiopathology of phosgene, they did not affect pulmonary edemagenesis [75, 77].

Thus, it appears that stimulation of pulmonary receptors not only may play a role in the control of breathing but may also affect heart rate (Fig. 2). This came as no surprise, as apnea may trigger a decrease in systemic vascular resistance upon severe acute stimulation of receptors [78]. Accordingly, the activation of nerve afferents—either by chemical irritants or by physical stresses—may have elicited the respiratory and cardiovascular reflex responses shown in Figs. 1 and 2 [78–82]. This striking coherence was also demonstrated by the increased Penh proportional to the length of the apnea period (Figs. 1, 2) and bradycardia (Fig. 2). Both events occurred during exposure to phosgene and remained remarkably stable during the ≈20-h post-exposure period, i.e., a period ranging from normal conditions to fully developed lung edema.

Li et al. [42] hypothesized that nociceptive C-fiber nerve endings may play a role in detecting the onset of pathophysiological conditions at the alveolar level. The afferent activity arising from these vagal nerve fibers also plays an important role in regulating cardiopulmonary function under both normal and abnormal physiological conditions [78]. Hence, the activation of these afferents by phosgene may elicit both respiratory and cardiovascular reflex responses. The hallmarks of this parasympathetic stimulation were believed to be linked to prolonged apnea periods and bradycardia, as illustrated in Figs. 1 and 2. More recent research on ion channels of the transient receptor potential (TRP) family has identified that these receptors act as specific chemosensory molecules in the respiratory tract in the detection and control of adaptive responses and in the initiation of detrimental signaling cascades upon exposure to various toxic inhalation hazards, including phosgene. The TRP channel mechanism was considered a potential target for intervention in phosgene-induced ALI/ARDS [19, 83, 84].

### Analysis of biomarkers of pulmonary irritation and associated lung edema

**Fig. 3:**
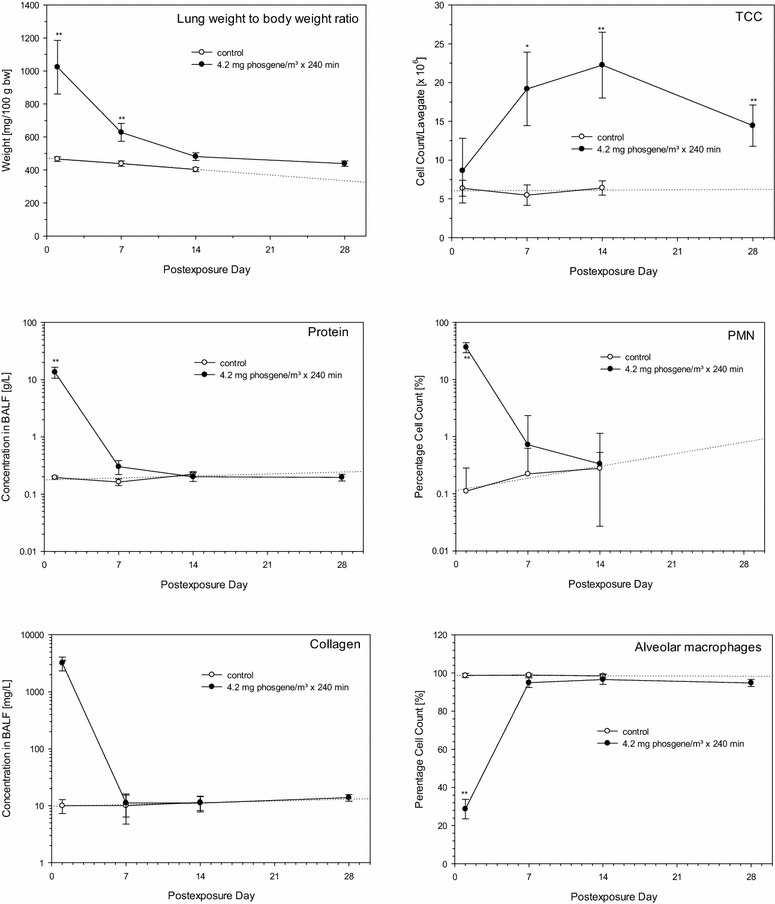
The *left column* compares time-course changes of endpoints suggestive of pulmonary edema (protein and soluble collagen in BALF, wet weight of excised lungs). Associated changes in cellular endpoints (TCC, PMN and alveolar macrophages in BALF) are shown in the *right column*. Rats were exposed to air (control) or phosgene at the non-LCt_01_ of ≈1000 mg/m^3^ × min. Time-course changes were examined by serial sacrifices on post-exposure days 1, 7, 14, and 28 (phosgene only). Data points represent means ± SD (six rats per group and time point). *Asterisks* (*) denote significant differences to the time-matched air control group (*P < 0.05, **P < 0.01)

Rats with nose-only exposure to phosgene at ≈LCt_01_ were used to analyze time-course changes in BAL indicative of acute pulmonary edema. Measurements began at the climax of the pulmonary edema (post-exposure day 1) and continued through 4 weeks post-exposure. Control data were collected from time-matched controls during the first 2 weeks (from which 4-week reference data were extrapolated, as illustrated in Fig. 3). The weight of excised lungs from exsanguinated rats was used as an all-integrating endpoint of ALI.

Lung weights, collagen and total protein in BALF, as surrogate endpoints of extravasated capillary fluid into the alveolar compartment accessible by BAL fluid, followed a similar trend (Fig. 3). The most sensitive endpoint was soluble collagen, followed by protein (Fig. 3). These biomarkers suggest that the alveolar barrier function appeared to be functionally restored on post-exposure day 7. The nonsignificantly higher lung weights relative to the control were attributed to increased adaptive activity and hypertrophy of type II pneumocytes. These cells are known to be engaged in both the removal of excessive fluids and surfactant synthesis as well as acting as stem cells for the restoration of damaged type I cells. Increased tolerance following single phosgene exposure [85] and studies with longer post-exposure periods support this conclusion [38].

With regard to the cellular components of BALF, total cell counts in BALF (TCC) increased significantly on post-exposure days 7 and 14 (Fig. 3). Cytodifferentials revealed conspicuously decreased alveolar macrophages (AM) 1 day post-exposure [20, 38]. The loss of AM appeared to be compensated by a marked influx of neutrophils (PMN), which were cleared from the lung as rapidly as the extravasation marker in BALF (Fig. 3). These findings show that an exposure dose of phosgene at the LCt_01_ may have caused a transient loss of functional alveolar macrophages with a concomitant loss of anti-microbial capacity. Concomitantly, chemotactic factors discharged from necrotic macrophages may have triggered the influx of neutrophils as a compensatory response. Altogether, this series of events suggests that PMNs temporally assumed the role of phagocytes with minimal or absent priming toward inflammatory cells. Phagocytes (TCC) were apparently engaged in the removal of dysfunctional surfactant and/or cellular debris over a period of several weeks.

### Interrelationship of hemoconcentration and increased lung weight

**Fig. 4:**
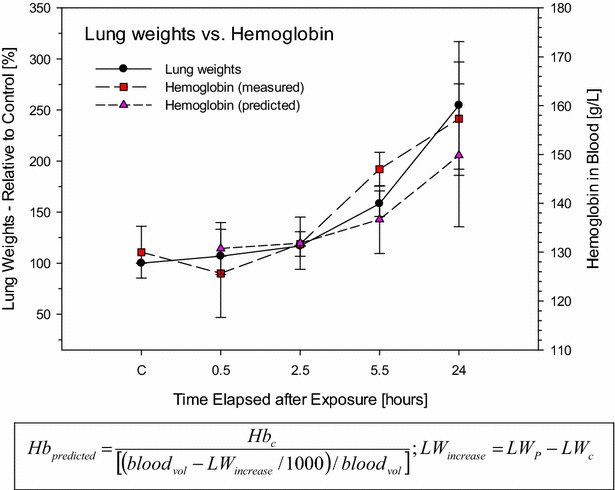
Association of time-related increased lung weights (LW) and hemoglobin (Hb) in blood to analyze interrelationships that could explain fluid-shifts from blood into the lung. The degree of hemoconcentration was predicted based on the gain of lung weights (LW_increase_) [mg] of phosgene-exposed rats relative to the lung weights (LW_c_) and hemoglobin (Hb_c_) [g/L] of pooled control rats. The blood volume was calculated using the following relationship: blood_vol_ [mL] = body weight [g] × 6.4 [%] [142]. As an approximation, increased lung weights were considered equal to increased lung water content. Data points represent means ± SD (three rats per group and time point)

The time-course changes in increased lung weight relative to those in hemoglobin (Hb) in blood after the exposure of rats to either air (control) or phosgene are compared in Fig. 4. Although originating from entirely different compartments, a coherent increase in either endpoint occurred up to the climax of pulmonary edema. Thus, the gain in lung weight relative to the control rats paralleled the loss of plasma fluid volume from the systemic circulation indicated by increased Hb. Progressive increases in Hb and lung weight occurred 5–6 h post-exposure. With increased time elapsed, the calculated Hb concentration was slightly lower than its measured concentration. This underprediction could be attributed to that fraction of accumulated fluid volume possibly being cleared from the lung into the lymphatic/pleural system at the later time points. This interpretation is substantiated by observations from acute inhalation studies of rats, which showed both pulmonary edema (trachea with white foamy content) and pleural effusions (hydrothorax) [37]. Moreover, minimal additional shift of plasma fluid into splanchnic organs cannot entirely be excluded.

This analysis provides unequivocal evidence of the redistribution of plasma fluid and proteins from the peripheral circulation into the lung. This pattern of changes is not peculiar to phosgene poisoning since similar findings were noted following exposure to other lung irritants [33]. Bradycardia and decreased cardiac output along with systemic vasoconstriction may have caused the redistribution of plasma volume into the lung. This process may have aggravated the acute edema and anoxic anoxia in the accompanying hemodynamic state of increasing hemoconcentration and blood viscosity. All of these factors, including those caused by intense vagus stimulation [82, 86, 87], seriously impede gas exchange and further lead to imbalances in the fluid dynamics of the lung. Collectively, cardiovascular disturbances (cardiogenic edema caused by imbalanced Starling forces), rather than an appreciable disruption of the air-blood barrier function, were believed to be the predominant etiopathology of the phosgene-induced lung edema (at this Cxt). Evidence from studies on larger animals and human evidence (military and occupational) report a similar interrelationship of hemoconcentration and pulmonary edema [54, 75, 76].

### Prognostic biomarkers in expired gas

**Fig. 5:**
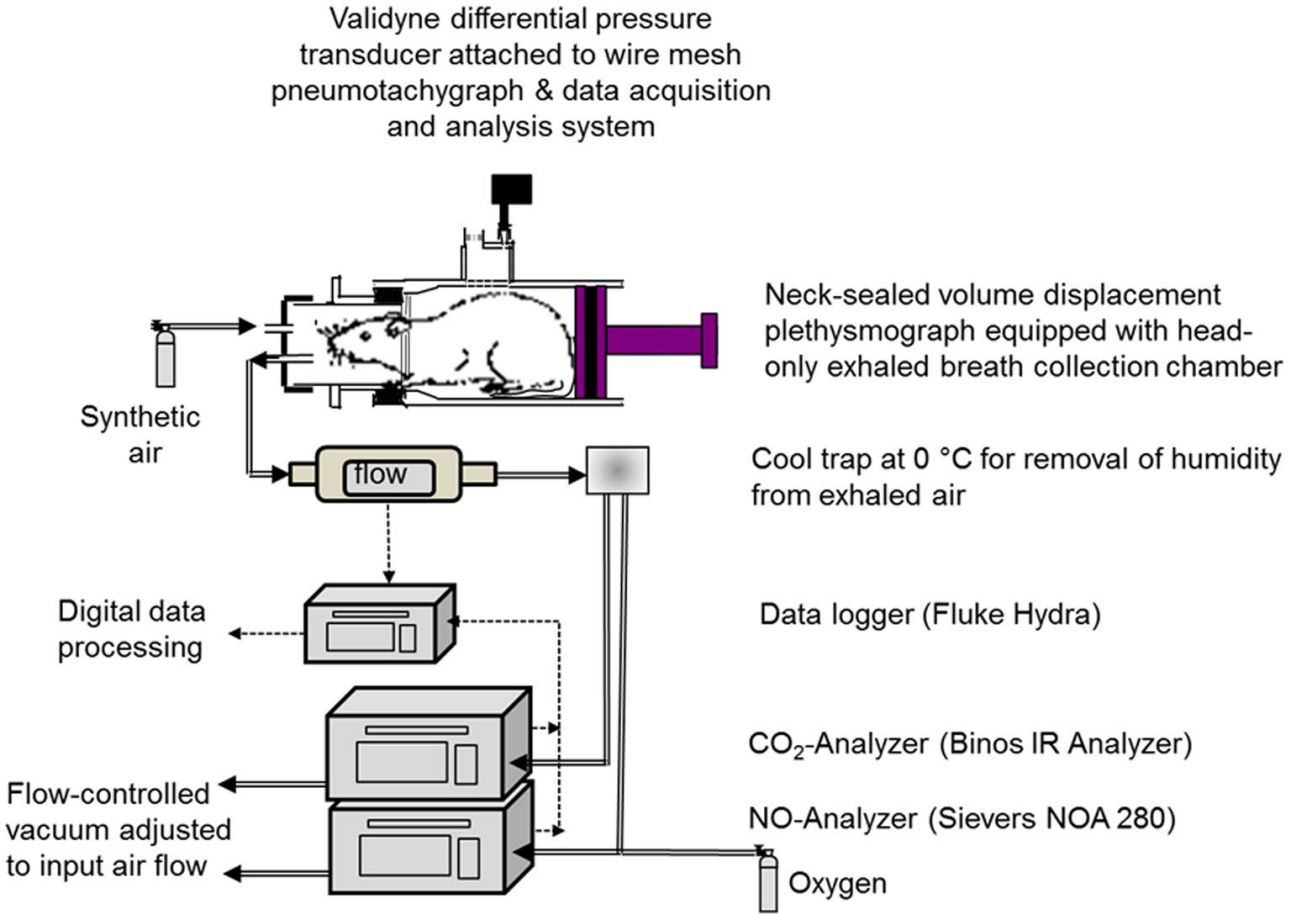
Schematic of the experimental arrangement to measure eNO, eCO_2_ and breathing frequency in spontaneously breathing, conscious rats. Rats were placed into a two-compartment restraining tube for data collection periods of 10 min (thoracic compartment: volume displacement plethysmograph; head-out compartment: bias-flow of synthetic air with manifold to the NO-chemiluminescence and infrared CO_2_-gas analyzers connected to a mass-flow controlled vacuum). ‘Flow’: mass flow meter/controllers. *Dotted lines* electrical connections, double lines: ducts for analyses in expired gas

A wealth of published evidence supports the prognostic relevance of measurements of physiological dead space (V_D_) relative to tidal volume (V_T_) for patients with ARDS [27, 28]. The value of V_D_/V_T_ measurements in predicting mortality in patients has been reaffirmed by several studies [29, 30, 88–90]. The specific value of measuring V_D_/V_T_ to increase the understanding of the pathophysiology of ARDS is based on the relatively high diffusibility of carbon dioxide across tissue membranes compared to oxygen [91]. Thus, V_D_/V_T_ is considered a more perfusion-sensitive variable that may be useful as an indirect marker of pulmonary endothelial injury [87]. Duplication of this assay was attempted in rats (Fig. 5) with consideration of the following limitations: (1) rats are uncooperative, which precludes forced maneuvers to measure end-tidal CO_2_ and nitric oxide (NO) in expired gas (eNO) and (2) the V_T_ and breathing frequencies of conscious, spontaneously breathing rats are in the range of 1–2 mL and 100–200 breaths/min, respectively, which requires additional sheath air to overcome the limitations of the dead spaces of apparatus and ducts, as detailed elsewhere [43].

**Fig. 6:**
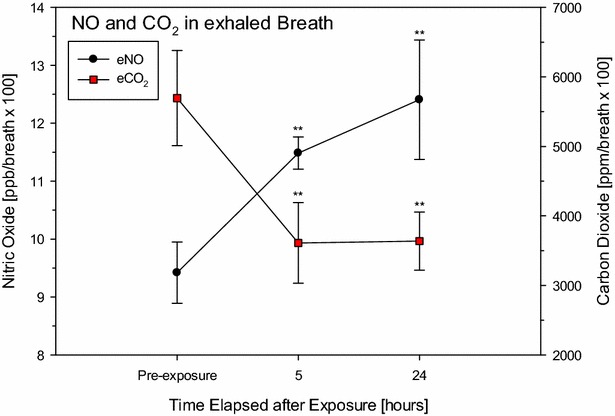
Measurement of exhaled eNO and eCO_2_ of rats 5 and 24 h post-phosgene exposure (for details see [43, 44, 46]). Sham control rats (denoted pre-exposure) served as concurrent control. Exhaled NO, CO_2_, and respiratory rate were digitally recorded every 10 s over a time period of 10 min. Data points represent means ± SD (n = 3). Values were normalized to 100 breaths. *Asterisks* (*) denote significant differences to the air control group (**P < 0.01)

Another limitation is that measurements of arterial CO_2_ tension (PaCO_2_) are more difficult to perform under such experimental conditions in rats compared to humans [92]. Thus, the method devised cannot be directly equated with volumetric capnography and ventilation dead space calculations, as suggested by Bohr [93] or Enghoff [94]. Indeed, measurements of FCO_2_ alone may not be sufficient to fully elucidate the relative contributions of venous admixture (shunt) and dead space [95]. Consistent with human data, eCO_2_ persistently decreased by more than 50% post-exposure (Fig. 6). A statistically significant increase in eNO occurred during the asymptomatic phase and the development of lung edema.

NOS-2 inhibitors are highly efficacious in the development of phosgene-induced ALI, especially when delivered by the inhalation route [96, 97]. Data from rats (Fig. 6) demonstrated that this non-invasive and readily available biomarker has the potential to deliver important prognostic information that could guide clinicians on countermeasures following accidental exposures to phosgene and other irritants [42, 43, 46, 47]. NO is considered an important mediator of acute lung injury (ALI) and is endogenously produced by NO synthase 2 (NOS-2), an enzyme upregulated in both ARDS patients and animal ALI models [98–100]. Recent studies have demonstrated that NOS-2 is induced in rat lungs exposed to phosgene [96, 101]. Hence, contemporaneous measurements of NO were thought to be an invaluable adjunct to exhaled CO_2_, as they may enable an integrated appreciation of the localized modulation of vascular tonus by NO suggestive of perfusion: ventilation imbalances.

**Fig. 7:**
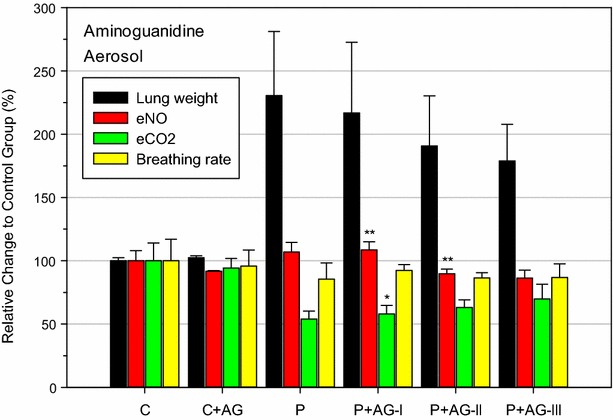
Impact of the inhaled NOS-inhibitor AG-aerosol on the phosgene-induced changes in lung weights, eNO, eCO_2_, and breathing frequency. AG (dissolved 7% in deionized water) was aerosolized to be inhalable for rats [44]. Control rats were nose-only exposed for 30 min to dry air (C). Rats from all other groups were similarly exposed for 30 min to phosgene (≈900 mg/m^3^ × min). AG groups were exposed to AG-aerosols in three modifications: (I) 300 mg drug/m^3^ for 30-min (nose-only, b.i.d. 0.5 and 5 h post-exposure to phosgene). (II) Same treatment regimen as (I) but animals were exposed unrestrained in small whole-body exposure chambers. (III) Same treatment regimen as (II) but the same inhalation dose was continually delivered over 6 h at 50 mg drug/m^3^. Hence, all groups received the same Cxt of drug. All endpoints were determined approximately 20 h post-exposure to phosgene. *Bars* represent means ± SD (n = 5). *Asterisks* denote significant differences of AG-groups relative to the phosgene (P) group (*P < 0.05, **P < 0.01)

In the proof-of-concept study shown in Fig. 7 [44, partially published], changes in these biomarkers in expired gas were systematically examined using different inhalation regimens at equal Cxts of aminoguanidine (AG) aerosol, a selective NOS-2 inhibitor: There was an unequivocal coherence of increased lung weights and decreased eCO_2_, which was partially reversed by AG aerosol treatment. While superimposed immobilization stress reduced the efficacy of the drug, non-immobilized animals in small whole-body chambers continually exposed to a lower AG concentration but for a longer duration (same Cxt of drug) showed visible improvements in lung weights and eCO_2_. The mild increase in phosgene-induced eNO was most favorably reduced under the AG-III regimen. This outcome demonstrated a definite interrelationship of phosgene-induced “occult” lung edema and increased ventilation dead space. Similar relationships were also observed in ARDS patients [29, 88, 102].

### Comparison of indices of ALI in rats exposed to phosgene or chlorine

The clinical consequences of accidental, high-level exposure to either chlorine [16, 39, 103–112] or phosgene gas [5, 34, 76, 113–115] have been well described. The objective of this comparative analysis was to compare phosgene, a poorly water-soluble alveolar irritant gas, with chlorine, a highly water-soluble airway irritant gas, at estimated equitoxic Cxts, which was 413 ppm × min for chlorine [47, 116].

**Fig. 8:**
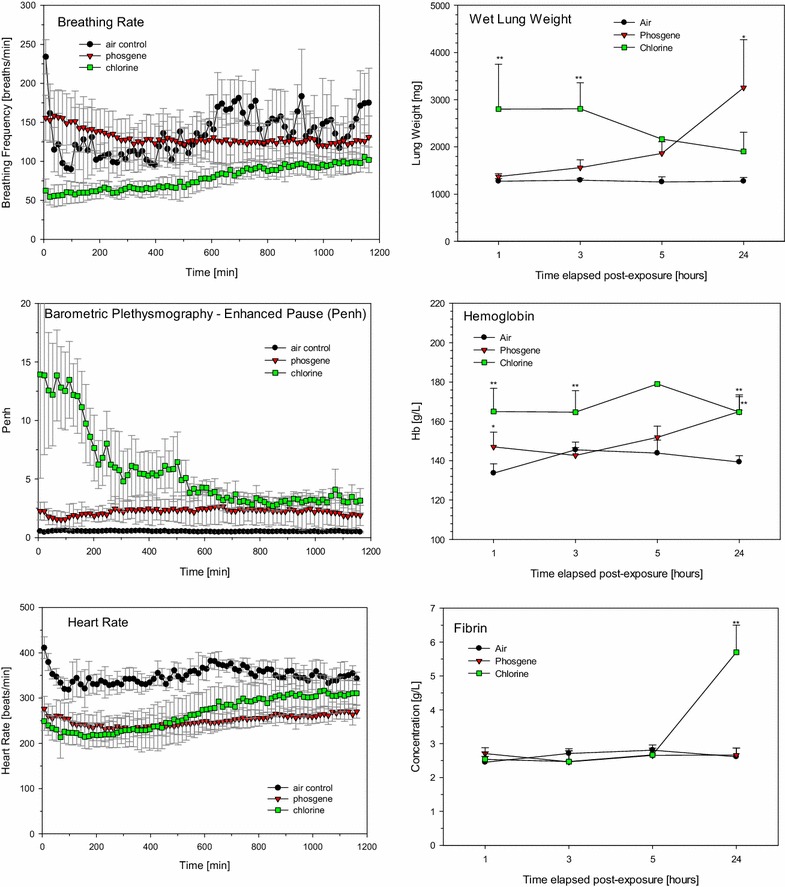
Comparison of three groups of rats sequentially nose-only exposed for 30 min to air, ≈32 mg/m^3^ (≈8 ppm) phosgene gas, or 197 mg/m^3^ (413 ppm) chlorine gas. Cardiopulmonary endpoints were determined as detailed in the caption of Fig. 2. Lung weights, hemoglobin, and fibrin were determined 1, 3, 5, and 24 h post-phosgene exposure (for details see [47]). Data points represent means ± SD (n = 6; however, due to unscheduled deaths in the chlorine group the actually examined number of rats were 3, 1, and 4 at the 3, 5, and 24 h sacrifices, respectively. *Asterisks* (*) denote significant differences between the phosgene and chlorine groups (*P < 0.05, **P < 0.01)

The lung weights of chlorine-exposed rats peaked 1-h post-exposure with partial resolution after 5 and 24 h. Opposite time-course changes occurred in phosgene-exposed rats (Fig. 8). Changes in Penh reflected the marked upper airway irritation (reflex bradypnea from trigeminal stimulation in the nasal passages with decreased breathing frequency) in chlorine-exposed rats. The alveolar irritant phosgene produced a much milder response (reflex apnea by J-receptor stimulation in the lower airways with minimal changes in breathing frequency). These typical periods of upper/lower respiratory tract irritation are considered ‘expiratory time’ by Penh. Heart rate depression (bradycardia) was almost indistinguishable between phosgene- and chlorine-exposed rats. Despite the more severe toxicological outcome in chlorine-exposed rats, bradycardia decreased more completely relative to the phosgene-exposed rats. Hb increased with time elapsed in phosgene-exposed rats, whereas a somewhat instant increase occurred in the chlorine-exposed animals. Fibrin was significantly elevated after 24 h in chlorine-exposed rats (Fig. 8). Phosgene-exposed rats were indistinguishable from the control. Enhanced intra-pulmonary fibrin deposition due to abnormal bronchoalveolar fibrin turnover and coagulopathy has been shown to be a hallmark of acute respiratory distress syndrome (ARDS) [103] and animal models [117–119]. Delayed onset of death occurred in rodents exposed to chlorine by mucus plugs and overshooting fibro-proliferative inflammation and regeneration [116], while delayed lethality did not occur in more recent studies of phosgene in rats [38]. The key findings highlighting the differences of phosgene and chlorine are summarized in Table 1.

**Table 1:** Salient markers of acute respiratory tract injury of phosgene and chlorine in rats

	Phosgene	Chlorine
Subjective symptoms	Absent	Eye and airway irritation
Sensory irritation-URT	Absent	Marked
Bronchial airway injury	Minimal, if any	Marked
Surfactant deterioration	Marked	Dose-dependent
Sensory irritation-LRT	Marked	Dose-dependent
Alveolar macrophage injury	Marked	Dose-dependent
Pulmonary vascular dysfunction	Marked	Dose-dependent
Cardiopulmonary dysfunction	Marked	Marked
Early lung edema	Extreme doses	Dose-dependent
Onset of lung edema	Maximum 15–20 h	Instant
Primary countermeasure	Lung edema	Lung edema & obliterating airway injury
Secondary countermeasure	Rapid recovery	Lingering airway injury
Clinical guidance on inhaled dose	Phosgene dosimeters	Environmental analyses (if available)
Prognostic approaches	Hemoglobin, eNO, eCO_2_	Irritation severity, fibrin

*URT* upper respiratory tract, *LRT* lower respiratory tract, *eNO* exhaled nitric oxide, *eCO*_*2*_ exhaled carbon dioxide

An analysis of protein and cytodifferentiated total cell counts in BALF one day post-exposure to chlorine or phosgene revealed identical decreases in total cell counts, with slightly lower counts of alveolar macrophages in the phosgene-exposed group. Protein in BALF was minimally elevated following exposure to chlorine and was maximally increased after exposure to phosgene. Exhaled breath analyses showed decreased eCO_2_ after either gas but higher eNO in chlorine-exposed rats, especially on the day of exposure [47].

## Inhalation studies—larger animals

Evidence collected from studies in small animals supports the conclusion that phosgene-induced pulmonary edema was initiated and propagated by events that affect the lungs’ overall control of lung mechanics and was indirectly initiated by tissue congestion and decreased vascular compliance/airway patency. Secondary changes in cardiovascular control and hemodynamics add another dimension of complexity. These conclusions from small-animal models were further substantiated in exploratory proof-of-concept studies in dogs [17] and pigs [22] with protective positive PEEP ventilation. This mode of ventilation strategy was demonstrated to improve survival in patients with ARDS [31, 120, 121], especially when begun early after the initiating encounter. Dogs were exposed at clearly lethal levels (135 mg/m^3^ × 25 min, equivalent to 3350 mg/m^3^ × min or 820 ppm × min). Shortly after exposure, the dogs were anesthetized, intubated and then subjected to PEEP (V_T_ = 10–12 mL/kg body weight, 40 breaths/min; FiO_2_: 0.21) at 0, 4, or 12 cm H_2_O over a post-exposure period of 8 h (one dog per setting). The lung edema was markedly alleviated at 4 cm H_2_O, but not at 0 cm H_2_O of PEEP. Microscopy confirmed reduced hemorrhage, neutrophilic infiltration, and intra-alveolar edema. Thus, the time-dependent progression into life-threatening pulmonary edema can be effectively suppressed by protective, low-pressure PEEP when implemented sufficiently early after exposure to phosgene [17]. Anesthetized pigs were instrumented and exposed to phosgene (concentration × time (Cxt), 2350 mg × min/m^3^ or 573 ppm × min) and then ventilated with intermittent PEEP (V_T_ = 10 mL/kg; PEEP 3 cm H_2_O; 20 breaths/min; FiO_2_: 0.24), monitored for 6 h after exposure, and then randomized into treatment groups: V_T_ at 8 or 6 mL/kg; PEEP 8 cm H_2_O; 20 or 25 breaths/min; FiO_2_: 0.4. This study aimed to examine the pathophysiological changes observed with low-V_T_-protective ventilation strategies compared to conventional ventilation. Pathophysiological parameters were measured for up to 24 h. The results showed improved oxygenation and decreased shunt and mortality, with all the animals surviving to 24 h compared to only three of the conventional ventilation animals. Microscopy confirmed reduced hemorrhage, neutrophilic infiltration, and intra-alveolar edema [22].

From phosgene inhalation studies in dogs at 1880 ppm × min (7708 mg/m^3^ × min), it was concluded that, under the given experimental conditions, immediate therapy with O_2_ is vital and FiO_2_ of 0.4–0.5 is sufficient [25]. Timely correction with NaHCO_3_ infusion was recommended for base deficit; however, the associated negative consequences must be thoughtfully considered (for details, see ‘permissive hypercapnia’ below). There was no apparent benefit from cortisone, theophylline, PGE_1_ or atropine. Jugg and coworkers published a more comprehensive comparison of large animal models using therapeutic approaches [9, 25, 26].

## Improved recognition of high-risk patients and triage

As exemplified for phosgene, the most critical phase for prognostic triage and successful preventive treatment is the asymptomatic, rather than the symptomatic phase. The comparison of the predominantly airway irritant chlorine with the alveolar irritant phosgene demonstrated appreciable differences in injury patterns. This result justifies not only different countermeasures but also the appropriate diagnostic tools to guide optimal treatment. Elevated concentrations of fibrin and hemoglobin in blood as well as CO_2_ and NO measured in expired gas were shown to be practicable and sensitive biomarkers of site-specific injuries within the respiratory tract. Re-triage by time-course measurements of CO_2_ and NO in exhaled breath using protocols distinguishing the fraction of breath from the airways and alveoli may increase the diagnostic power of this assay [92, 122]. Bedside quantification of dead space could be used to titrate countermeasures at the asymptomatic stage of injury. In cases of exposure to mixtures of irritant gases, late complications cannot be entirely excluded. Therefore, prior to discharge of patients or before changing treatment strategies from anti-edema to anti-inflammatory, these readily available analyses may deliver important information to clinicians regarding which course to take. These methods appear to be easy to manage and suitable for both triage and re-triage.

## Prevention strategies

Commonly, practitioners and clinicians alike are guided by the symptoms elaborated in putatively exposed subjects for the identification of high-risk patients. Most often, treatment follows reactive rather than proactive approaches, with an emphasis on treating rather than preventing the progression of worsening lung injury. Frequently, countermeasures appear to focus on PaO_2_ or saturation [32] to determine whether treatment strategies are effective. However, PaO_2_ may not be an accurate surrogate of alveolar stability; therefore, reliance on PaO_2_ as a marker of lung function presumes that there is no self-perpetuating and progressing occult ALI leading to alveolar instability and eventually lethal edema. As shown by the preventive PEEP applied to dogs and pigs, there is evidence that oxygenation as a method to optimize PEEP is not necessarily congruent with the PEEP levels required to maintain an open and stable lung [31, 32]. Thus, optimal PEEP might not be personalized to the lung pathology of an individual patient using oxygenation as the physiologic feedback system. Likewise, non-personalized, unreasonably high PEEP pressures may block lymph drainage. Ideally, titration of PEEP by volumetric capnometry at low V_T_ appears to be the most promising strategy [92, 123]. Volumetric capnometry was shown to be helpful for monitoring the response to titration of PEEP, indicating that the optimal PEEP should provide not only the best oxygenation and compliance but also the lowest V_D_ while maintaining the V_T_ below a level that over-distends lung units and aggravates V_D_ and lung injury [92]. Thus, the improvements in oxygenation and lung mechanics after an alveolar recruitment maneuver appear to be better preserved by using injury-adapted PEEP than by any ‘one size fits all’ standardized approach. Notably, protective lung ventilation strategies commonly involve hypercapnia. Thus, permissive hypercapnia has become a central component of protective lung ventilatory strategies [121, 124–128].

## Pharmacological treatment

Due to the complex interactions among (patho)physiological events, it seems unrealistic to assume that any monocausal, drug-related treatment regimen will be identified in the near future to mitigate the particular type of ALI attributable to inhaled phosgene gas. This conclusion matches those of other authors [120, 129–131]. Collectively, the wealth of published evidence supports the conclusion that, if the acute stage of pulmonary edema with its attendant anoxic anoxia is survived, circulatory failure may become a more important factor in the ultimate outcome [65]. Likewise, a countermeasure identified to be efficacious for a non-water-soluble gas, such as phosgene, may not necessarily be the best countermeasure for a highly water-soluble airway and alveolar irritant gas, such as chlorine, and vice versa.

Multiple approaches for drug-related interventions, most of them anti-inflammatory and sympathomimetic, have been examined [9, 19, 22, 23, 25, 26, 55, 96, 132, 133]; however, none of these drugs have found their way into the clinic. To the contrary, as could be expected for phosgene, anti-inflammatory treatment with steroidal or non-steroidal drugs was either ineffective or even aggravated phosgene-induced ALI [21, 22, 44, 46]. More recent exploratory preclinical investigations have identified TRP inhibitors, NOS inhibitors, and statins as novel pharmaceutical approaches that prevent ALI; these drugs merit being studied in greater detail in the future [19, 31, 83, 84, 96, 134].

## Symptomatic or supportive treatment

As exemplified by many experimental studies in rats, an excess of water in the lung is not a consequence of too much water in the body; rather, it is a consequence of dysfunctional cardiovascular control to prevent excess fluid from accumulating in the septal interstitium and subsequent alveolar flooding. Hence, any use of diuretics may further aggravate the phosgene-induced hemoconcentration, rather than having any beneficial effect on the increasing pulmonary edema. Equally deleterious therapeutic results were obtained with bleeding or venesection (phlebotomy) and argue against these therapeutic options [65]. Notably, despite its vulnerable blood-air barrier, the lung is relatively resistant to the onset of pulmonary edema. This resistance is ascribed to several safety factors, which include increased lymph flow to drain fluids away from the lung and decreased interstitial oncotic pressure and interstitial compliance. These safety mechanisms are quite effective as long as surfactant prevents alveolar collapse [135–138]. The supine position increases gravity-related hydrostatic pressure and lung edema, which supports the prone positioning of patients [31]. The symptomatic treatment of hemoconcentration by non-conservative fluid resuscitation may change a non-lethal to a lethal lung edema, as this surplus fluid was shown to settle in the lung as edema [54, 139], as shown in previous dog inhalation studies with phosgene [65, 139–141]. Hence, fluid resuscitation should be handled most conservatively [115, 140]. The use of nebulized sympathomimetics may further contribute to reflexively induced changes in cardiac output and pulmonary hydrostatic pressure. Nebulized salbutamol treatment following phosgene-induced ALI did not improve survival and worsened various physiological parameters, including arterial oxygen partial pressure and shunt fraction. Anti-inflammatory corticoids have shown little benefit in patients with this type of cardiogenic lung edema in the absence of an inflammatory etiopathology. In summary, most of these types of “symptomatic treatments” might transform phosgene-induced ALI into iatrogenic ALI, rather paving the road to recovery [21, 25].

## Conclusions

Data from multiple animal species and mechanistic studies have coherently demonstrated that phosgene-induced ALI is unique compared to ALI induced by other, more water-soluble irritant gases. Phosgene-induced ALI is initiated with exposure and remains occult for hours post-exposure, depending on the dose inhaled. During this asymptomatic period, a range of reflex-related cardiovascular responses appears to be involved in triggering progressive changes in cardiopulmonary and hemodynamic homeostasis. This imbalance of neurophysiological control may progressively shift fluid from the peripheral to the pulmonary circulation, leading to potentially lethal alveolar edema. Any proposed therapies targeting the prevention or early treatment of lung injury prior to respiratory failure require triage to identify patients at high risk, as resources are limited. CO_2_ and NO in exhaled breath were shown to be prognostic for edema occurring hours later.

Most importantly, clinicians should refrain from non-rationalized or common symptomatic treatments that could accelerate the progression of ALI. Preventive and personalized treatment strategies of mechanical ventilation with feedback loops focusing on lung function and conservative fluid management should be given preference.

In summary, current knowledge about the sequelae of phosgene-induced ALI has clearly positioned the field to undertake steps toward preventive or causal treatment, rather than mere symptomatic treatment; however, much work and communication remain necessary to make therapies effective, practical, and safe for asymptomatic subjects. The objective of the course taken in this paper was to challenge the often-exercised ‘trial-and-error’ type of symptomatic treatment in the absence of any mechanistic understanding.

## Abbreviations

AG: aminoguanidine
ALI: acute lung injury
AM: alveolar macrophage
AOP: adverse outcome pathways
ARDS: acute respiratory distress syndrome
AT: apnea time
BAL: bronchoalveolar lavage
BALF: BAL fluid
BALC: BAL cells
b.i.d.: bis in die (twice a day)
C: control
Cxt: inhaled dose expressed as the product of exposure concentration x exposure duration
Cl_2_: chlorine
CO_2_: carbon dioxide
eCO_2_: concentration of CO_2_ in expired gas
ECG: electrocardiogram
eNO: concentration of NO in expired gas
ET: expiratory time
FiO_2_: fraction of inspired O_2_
HCl: hydrochloric acid
iNOS: inducible nitric oxide synthase
IT: inspiratory time
LCt_01_: time-adjusted lethal concentration at 1% mortality
LCt_50_: time-adjusted median lethal concentration
LW: lung weight
MV: respiratory minute volume
NaHCO_3_: sodium carbonate
NO: nitric oxide
NOAEL: no-observed-adverse-effect level based on experimental data
OECD: Organization for Economic Cooperation and Development
P: phosgene
PEEP: positive end-expiratory pressure
PGE_1_: prostaglandin E_1_
PIF: peak inspiratory flow
PEF: peak expiratory flow
PMN: polymorphonuclear neutrophil
POD: point of departure
PaCO_2_: arterial PCO_2_
Penh: enhanced pause
Rf: relaxation time
SD: standard deviation
TRP: transient receptor potential
TCC: total cell count
V_T_: tidal volume
V_D_: ventilation dead space
WWI: World War I
